# Aging impairs type 2 immune responses to nematodes associated with reduced gut microbiota responsiveness

**DOI:** 10.1038/s41598-025-16730-x

**Published:** 2025-08-26

**Authors:** Motoko Morimoto, Sota Tanaka, Kyoko Jinguji, Wakako Ikeda-Ohtsubo

**Affiliations:** 1 School of Food Industrial Sciences, Department of Food Resource Development, Miyagi University, Sendai, Miyagi 982-0215 Japan; 2 Laboratory of Animal Food Function, Graduate School of Agricultural Science, Tohoku University, Sendai, Miyagi 980-8572 Japan

**Keywords:** Type 2 immune response, Intestinal nematode infection, Short-chain fatty acids, Gut microbiota, Aging, Helminth, Infection, Parasitic infection, Microbiome

## Abstract

Gastrointestinal nematode infections elicit robust type 2 immune responses that facilitate rapid parasite expulsion. Our previous studies demonstrated that 18-month-old mice exhibit both impaired nematode clearance and reduced type 2-cytokine production, suggesting that aging diminishes the host’s potential to mount effective immune defenses. To further investigate the underlying mechanisms, we compared young (3 months old) and aged mice (18 months old) infected with the nematode *Heligmosomoides polygyrus* (Hp), focusing on the interplay between type 2 immune responses and intestinal ecology. Hp infected young mice exhibited increased expression of Th2 cytokines (e.g., *il-4*) and short-chain fatty acid (SCFA) receptors GPR41/GPR43, while these responses were markedly diminished in aged mice. Correspondingly, cecal SCFA levels—particularly acetate and propionate—increased in Hp infected young mice but decreased in aged counterparts. Moreover, Hp infection induced a pronounced shift in the cecal microbiota composition of young mice, notably a reduced Bacillota/Bacteroidota ratio (F/B) ratio, a change much less evident in aged mice. These findings suggest that the age-related decline in type 2 immune responses to gastrointestinal nematode infection is linked to reduced gut microbiota responsiveness, which may compromise host resistance to the gastrointestinal parasites.

**Supplementary Information:**

The online version contains supplementary material available at 10.1038/s41598-025-16730-x.

## Introduction

Aging accompanies deterioration of the immune system, e.g., the functional decline in T cells, which are often claimed to be a major cause of increased diseases risks^1,2^. Experimental and clinical studies have elucidated the mechanisms of age-related decline of immune system and it is now evident that immune cells experience varying levels of alteration by aging. The age-related decline of naïve T cells and increased proportion of ineffective memory cells cause impaired immune responses to infections by new pathogens^3^. The imbalance of functional immune responses including under- or over-expression of cytokines has been shown to activate NF-κB thereby enhance inflammatory responses in aged individuals, which is recently referred to as ‘inflammaging’^4,5^. In previous studies, we found that type 2 immune response induced by infection by gastrointestinal nematode parasites also experiences age-related decline^6,7^. The expression of type 2 cytokine genes was significantly reduced in 18-month-old aged mice, which caused impaired nematode expulsion ability^6,7^. Meanwhile, the number of CD4 + T cells and their accumulation remained at the same level as in the 3-month-old young mice, which suggested that the impaired ability of nematode expulsion in aged mice can be attributed to impaired activation of Th2 lymphocytes rather than their migration to the infection site. Indeed, an early study have demonstrated that aged mice exhibit impaired activation and function of Th2 cells (CD4 + T cells), which is associated with abnormal secretion of inflammatory cytokines^8,9^. Multiple lines of evidence indicate that aging induces functional defects in both naïve and memory CD4 + T cells, which leads to impaired type 2 immune responses and altered activation of these cells in aged individuals^10–12^. Nevertheless, there is limited evidence directly linking defects in Th2 lymphocytes to impaired type 2 immune responses in aged mice.

We assume that there might have been additional factors that connect between T cell function and type 2 immune responses, potentially preventing the appropriate activation of type 2 immune response against Hp infection in aged mice. The gastrointestinal tract, the primary infection site of gastrointestinal nematode parasites, harbors a substantial population of resident microorganisms that can affect host health in both beneficial and detrimental ways^13,14^. The composition and diversity of the gut microbiota significantly influence host metabolism and immune function, and the connections between the gut microbiota and aging are widely discussed^15,16^. Age-related shift in the gut microbiome can disrupt intestinal homeostasis and contribute to systemic inflammation^17,18^which may also affect the function of T cells and type 2 immune responses. Multiple studies have reported gut microbiota do respond to gastrointestinal nematode infection by changing their community composition in mice^19–21^. Also, the altered microbiota by nematode infection may result in increased amount of intestinal short chain fatty acids (SCFAs), which confer protective effects against allergic symptoms^22,23^. Nevertheless, although previous studies have examined the effects of aging on immune responses and gut microbiota, as well as the role of the microbiota in immune responses, the specific relationship between the age-related declines in immune responses to nematode infection and changes in the gut microbiota remains unexplored. Also, the impact of the microbiota on type 2 immune responses has been discussed in the context of aging^24,25^, direct evidence demonstrating this effect through comparisons between young and old populations remains lacking. To clarify this point, we compared profiles of type 2 immune responses and gut microbiota in young (3-month-old) and aged (18-month-old) mice upon infection by gastrointestinal nematode parasite *Heligmosomoides polygyrus* (Hp). Our current study shows age-related decline of type 2 immune response to intestinal nematode infection reflects altered intestinal ecology. Reduced responsiveness of the gut microbiota to gastrointestinal nematode infection was observed in aged mice, which likely caused the impairment of nematode clearance.

## Results

### Cytokine gene expression in the small intestine of Hp infected and noninfected young and aged mice

**Fig. 1:**
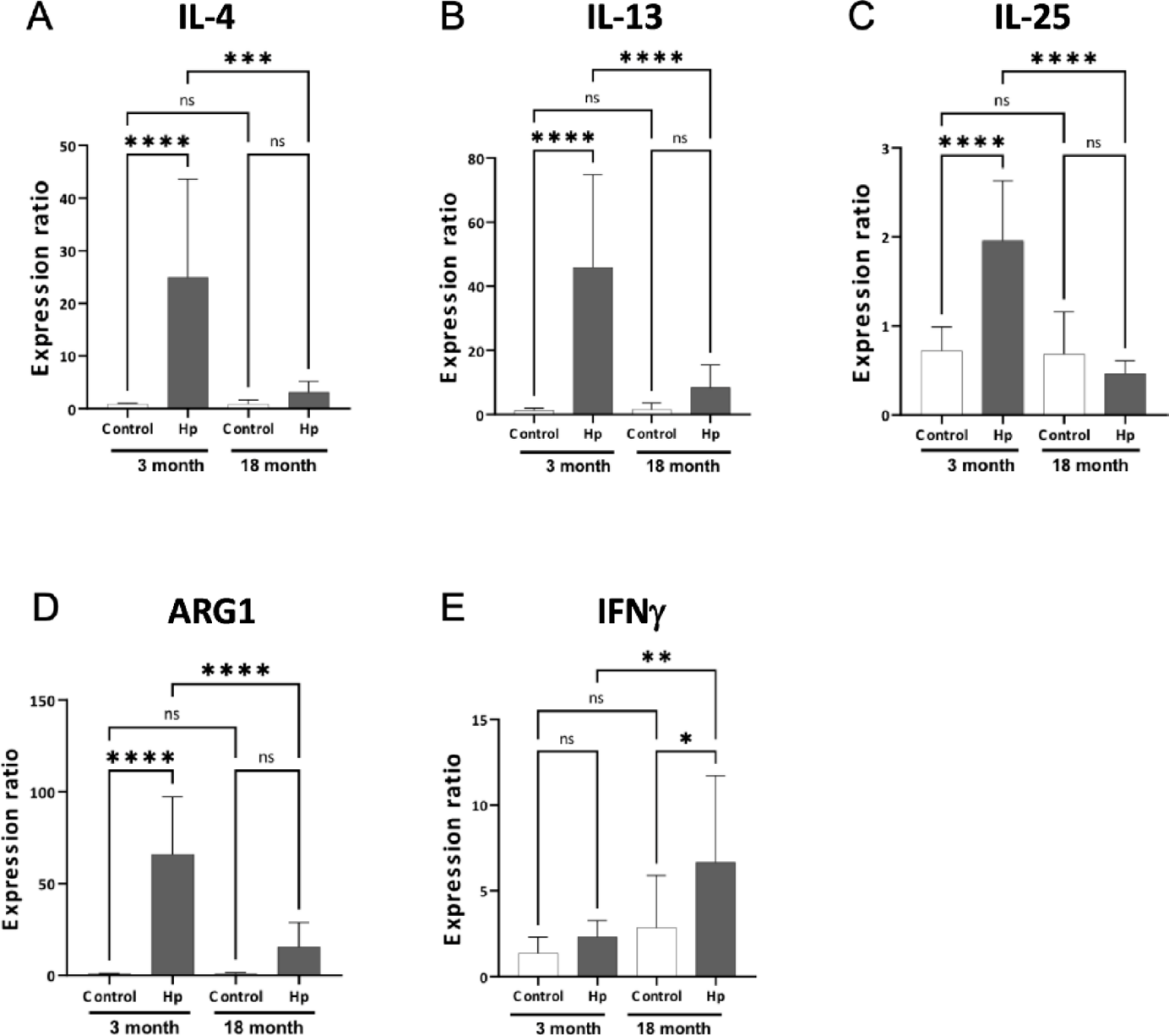
Real-time PCR analysis of cytokine and effector gene expression in the small intestine following *Heligmosomoides polygyrus* (Hp) infection. Expression levels of IL-4 (**A**), IL-13 (**B**), IL-25 (**C**), and ARG1 (**D**), representing type 2 immune response markers, and INFγ (**E**), representing a type 1 immune response marker were examined. Total RNA from whole intestinal tissues collected from uninfected control mice and from mice 8 days after Hp infection were used. Comparisons were made between young (3-month-old; white bars) and aged (18-month-old; black bars) mice (*n* = 10 per group). Statistical significance was determined using two-way ANOVA followed by the Tukey test as indicated in the Methods. *P < 0.05, **P < 0.01, ***P < 0.001, ****P < 0.0001; The data are expressed as the mean ± standard error (SE) from two independent experiments that yielded similar results.

We have previously reported the age-related decline in the Th2 cytokines IL-4 and IL-13, which are known to be induced by nematode infection^6^. In addition to IL-4 and IL-13, we investigated gene expression profiles of IL-25 and M2 macrophage marker arginase1 (Arg1), which have been shown to be involved in Th2 cytokine response against nematode infection^26,27^in Hp infected and non-infected young and aged mice. On day 8 after Hp infection, mRNA expression of IL-4, IL-13, and IL-25 genes was significantly upregulated in young (3-month-old) mice compared to their uninfected counterparts (Fig. 1A–C). In contrast, the elevation of gene expression level in response to Hp infection was not significant in aged (18-month-old) mice. Similarly, Arg1 gene was highly expressed in young mice upon Hp infection, but this upregulation was not prominent in aged mice (Fig. 1D). Meanwhile, the gene expression of IFNγ, which is responsible for upregulation of type-1 cytokines, showed an opposite profile where aged mice exhibited increased gene upregulation compared to the young mice after Hp infection (Fig. 1E).

### Cecal short-chain fatty acids (SCFAs) in Hp infected and noninfected young and aged mice

**Fig. 2:**
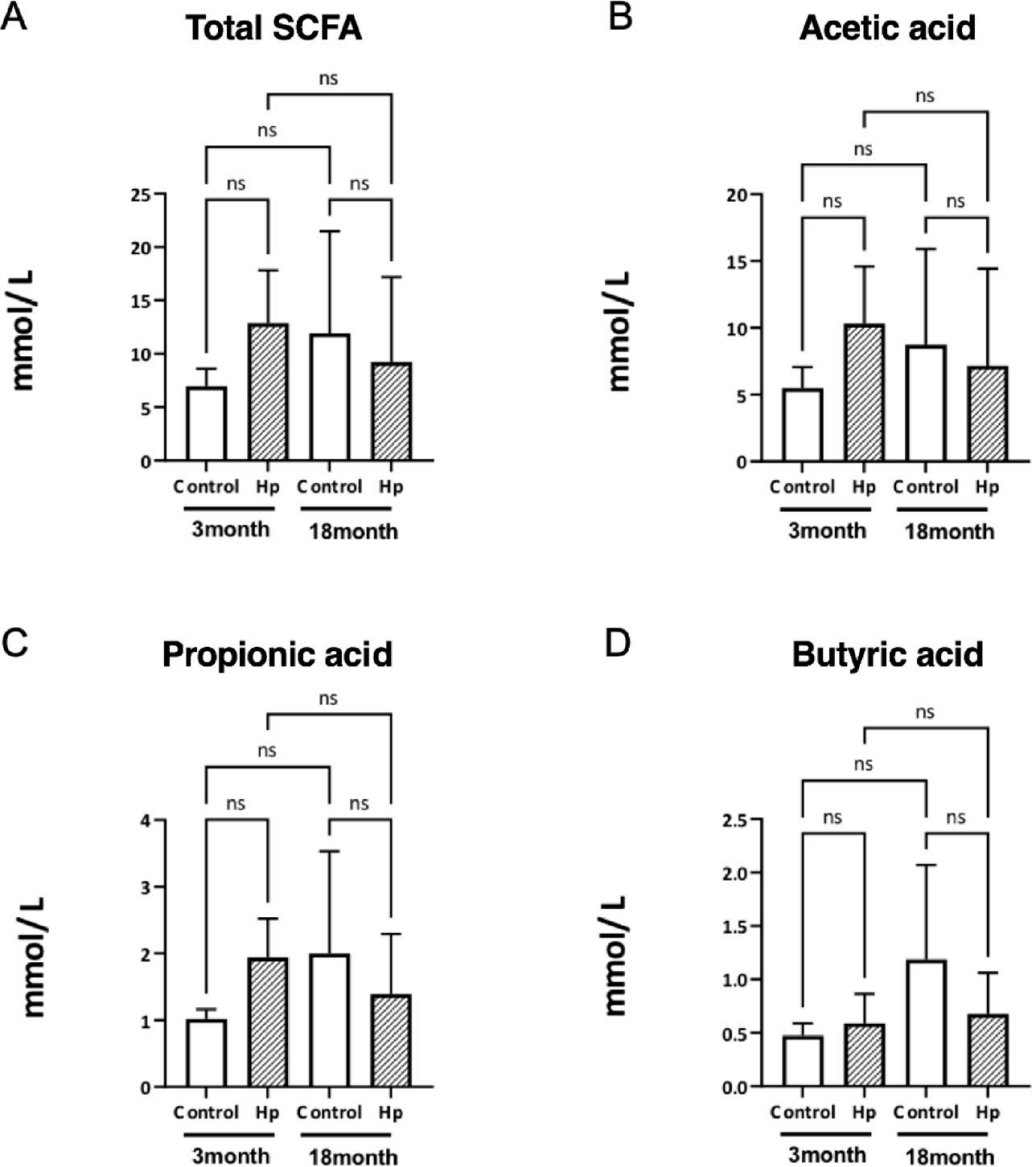
Amounts of short-chain fatty acids (SCFAs) in the cecal contents of *Heligmosomoides polygyrus* (Hp)-infected (diagonal stripe bars) and non-infected (white bars) young (3-month-old, 3 M) and aged (18-month-old,18 M) mice. (**A**) Total SCFAs, (**B**) acetic acids, (**C**) propionic acid, and (**D**) butyric acid were quantified using gas chromatography as described in the Methods. No statistically significant differences were observed (significance indicated by * *P* < 0.05 vs. control values).

SCFAs are major gut microbial metabolites that are known to regulate host immunity and metabolism via modulating T-cells^28,29^. As mentioned above, gastrointestinal nematode infection has been shown to increase SCFAs in mice^22,23^; it is therefore of interest to determine whether the SCFA production stimulated by nematode infection is affected by aging. We measured acetic acid, propionic acid, and butyric acid in the cecal contents and found that the SCFA concentrations exhibited age-related decline. While an elevated level of SCFAs was observed in young (3-month-old) mice in response to Hp infection, the level was lowered in aged (18-month-old) mice (Fig. 2A). This tendency was consistent across individual short-chain fatty acids: acetic acid (Fig. 2B), propionic acid (Fig. 2C), and butyric acid (Fig. 2D). Interestingly, SCFA levels showed a tendency to decrease in aged mice following Hp infection (Fig. 2A–D), although statistically not significant. Overall, the increased production of SCFAs in response to Hp infection appears to be diminished in aged mice.

### Changes in GPR41 and GPR43 expression in the intestinal tissue of Hp infected and noninfected young and aged mice

**Fig. 3:**
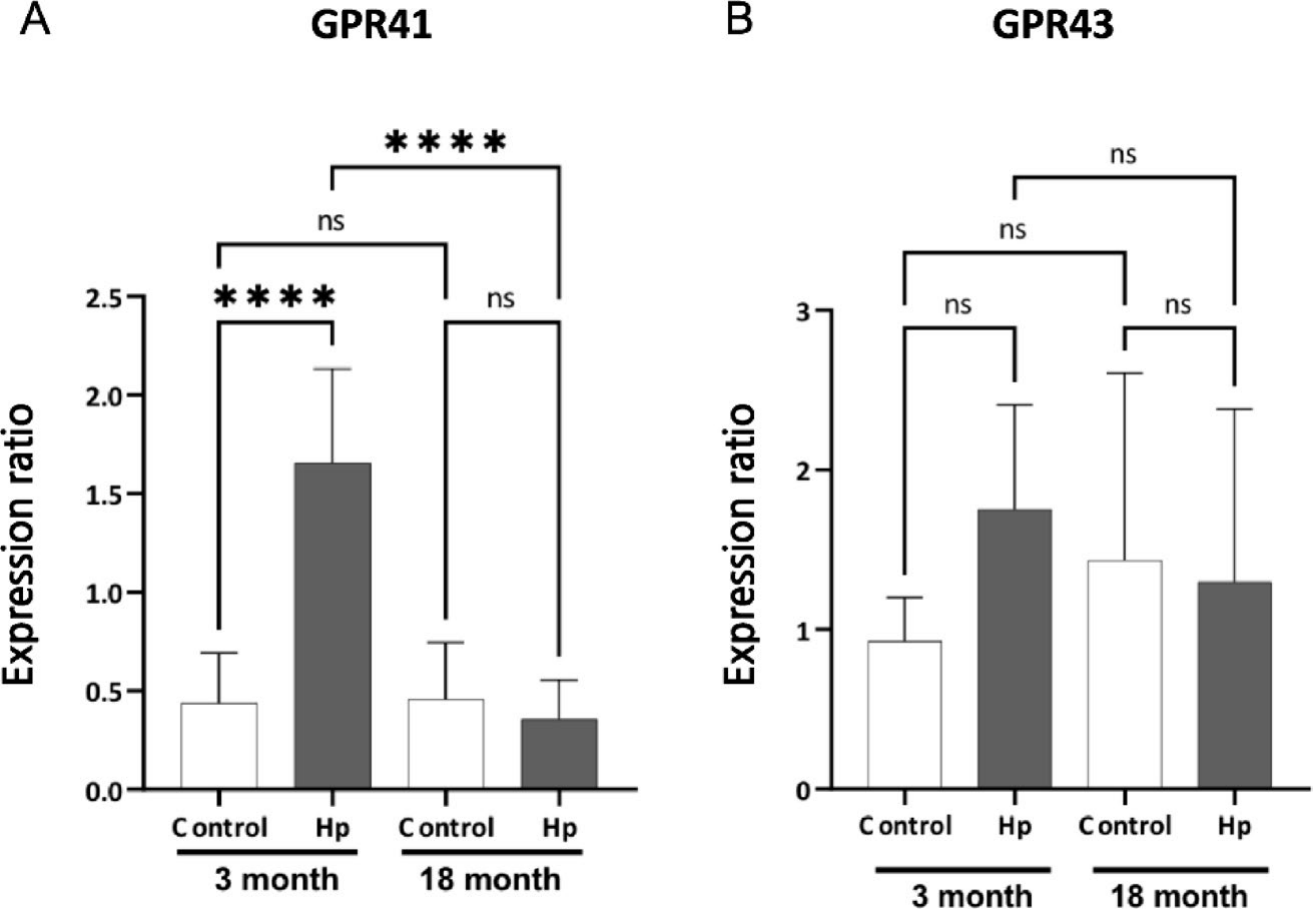
Gene expression of GPR41(**A**) and GPR43 (**B**), G-protein coupled receptors that sense short-chain fatty acids (SCFAs) were examined in whole intestinal tissues from uninfected control mice and from mice 8 days after *Heligmosomoides polygyrus* (Hp) infection. Comparisons were made between young (3-month-old; white bars) and aged (18-month-old; black bars) mice (*n* = 10 per group). Statistical significance was determined using two-way ANOVA followed by the Tukey test as indicated in the Methods and statistical significance is indicated ****P < 0.0001; The data are expressed as the mean ± standard error (SE) from two independent experiments that yielded similar results.

SCFAs produced by gut microbiota have been known to directly activate host G protein-coupled receptors, particularly GPR41 and GPR43, which induce the expression of chemokines and cytokines^30^. GPR41, also called FFAR3, is expressed on endothelial cells and enteric neurons^31^while GPR43, also called FFAR2, is expressed on adipocytes and immune cells^32^. We investigated how the expression of GPR41 and GPR43 in the small intestine was affected by Hp infection in young and aged mice. Gene expression of GPR41 was significantly increased in the Hp-infected young mice compared to the non-infected ones, but this up-regulation was not observed in Hp-infected aged mice. (Fig. 3A). A similar trend was observed for GPR43, although the differences were less pronounced than those seen with GPR41 (Fig. 3B). Collectively, the up-regulation of GPR41/43 expression in response to Hp infection was associated with the elevated levels of SCFAs in the cecal contents of young mice (Fig. 2), and these specific responses were no longer detectable in aged mice.

### Microbiota alteration in response to Hp infection in young and aged mice

**Fig. 4:**
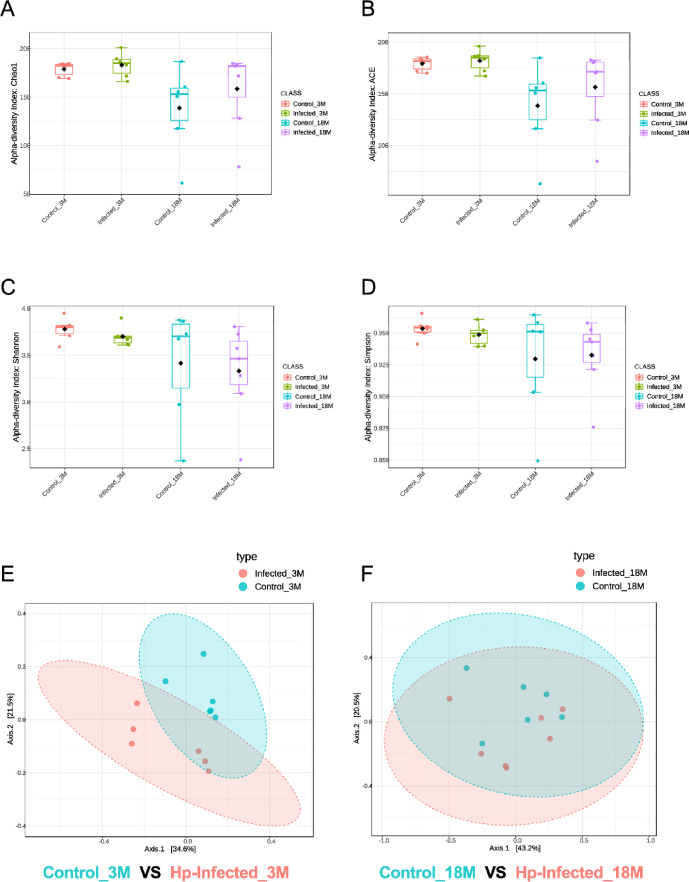
Species-level bacterial diversity in cecal contents of young (3 months, 3 M) and aged (18 months, 18 M) mice, *Heligmosomoides polygyrus* (Hp)- infected (Infected) and non-infected (Control) groups. (**A**,** B**) Alpha diversity: species-level richness assessed by Chao1 (**A**) and ACE (**B**) indices. (**C**,** D**) Alpha diversity: species evenness assessed by Shannon (**C**) and Simpson (**D**) indices. (**E**,** F**) Beta diversity: principal coordinate (PCoA) plots based on Bray-Curtis dissimilatory, comparing Control and Infected groups in young mice (E; 3 M, 3-month-old) and aged mice (F; 18 M, 18-month-old).

The lowered levels of intestinal short-chain fatty acids and their receptors (GPR41/43) in aged mice upon infection may indicate alterations in gut microbiota. Diversity analyses of cecal DNA from Hp-infected and non-infected young and aged mice, based on 16S rRNA gene sequencing, revealed differential responses of gut microbiota to Hp infection between the young and aged mice (Fig. 4). While read counts of infected groups were relatively higher than non-infected groups (Supplementary Fig. S1), bacterial species-level diversity and richness (alpha diversity) of cecal microbiota were not significantly affected by Hp infection in young and aged mice groups (Fig. 4A, B, C, D). As reported previously, aging—rather than Hp infection—significantly affected the alpha diversity, which is illustrated by the index showing that non-infected aged mice (light blue) had significantly decreased diversity compared to their young counterparts. (red; Chao1, *P* = 0.0799111; ACE, *P* = 0.072809). This age-related decrease of species-level richness was not as significant (*P* > 0.1) in Hp infected groups (green and purple; Fig. 4A, B). The species-level evenness (Shannon and Simpson) was consistent across the mice groups, but the individual variation was more pronounced in aged mice (*P* > 0.1; Fig. 4C, D).

Species-level analysis of beta diversity, which indicates the distinctness among communities, showed that the influence of Hp infection on cecal microbiota significantly differed between young and aged mice (Fig. 4E, F). In young mice, the distinction between the cecal microbiota of Hp-infected and non-infected mice was significant (PERMANIVA, *p* = 0.015; Fig. 4E), whereas this difference was not evident in aged mice (PERMANIVA, *P* = 0.353; Fig. 4F). Aging was found to increase beta diversity in both Hp infected and noninfected groups of mice (Supplementary Fig. S2), which is consistent with recent observations of age-related shifts in microbial composition in humans^33^.

### Relative abundance of bacterial groups responding to Hp infection

**Fig. 5:**
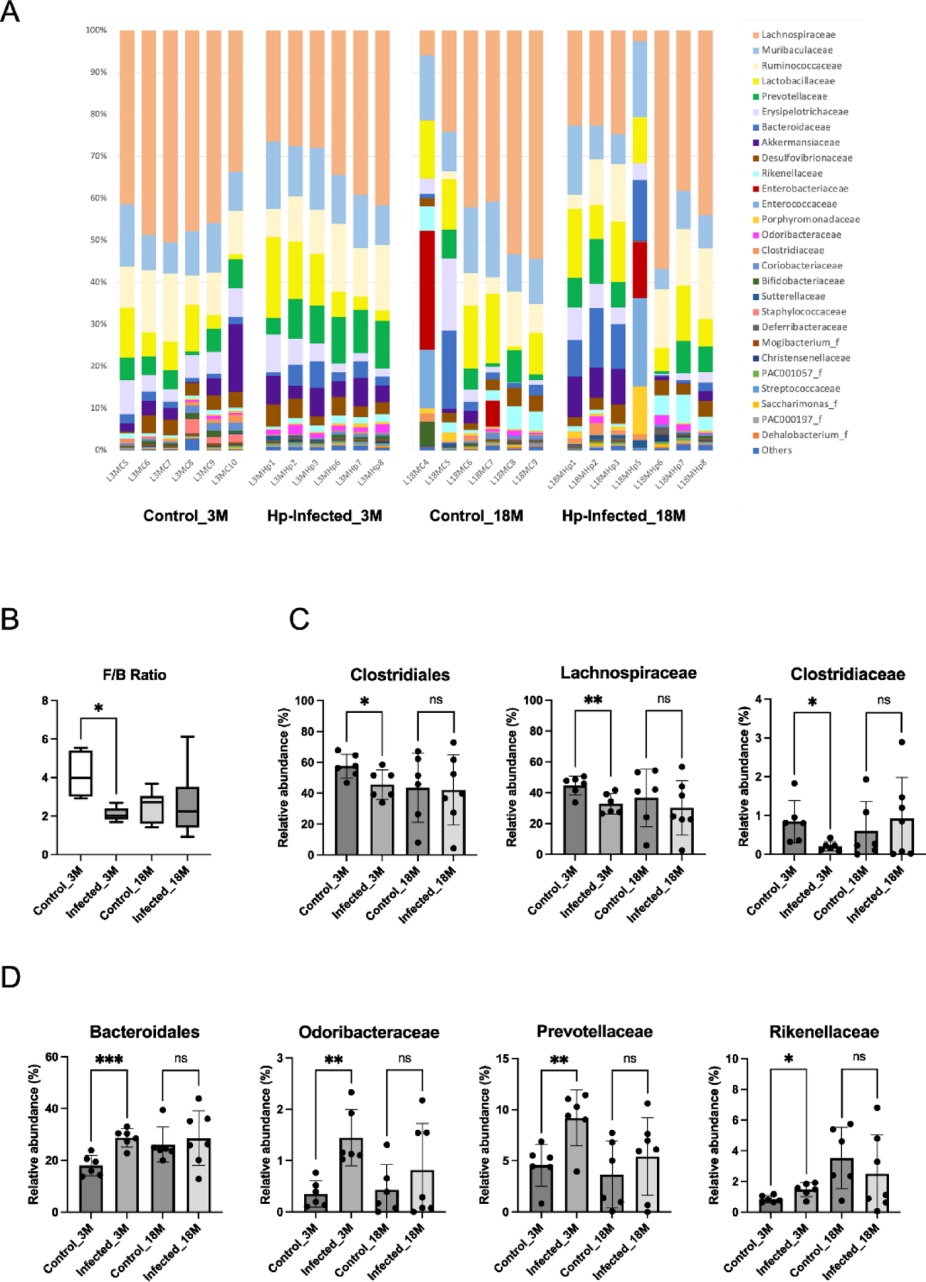
Comparison of relative abundance of representative bacterial taxa in mice cecal microbiota (**A**) Family-level bacterial composition in cecal microbiota of young (3 M, 3-month-old) and aged (18 M, 18-month-old) Hp infected (Infected) and non-infected (Control) mice groups. (**B**) The ratio of the bacterial phyla Bacillota and Bacteroidota (F/B ratio) of cecal microbiota of the four mice groups. (**C**) Relative abundance of bacterial order and families of the phylum Bacillota, which differed significantly between the Hp-infected and non-infected young mice groups. (**D**) Relative abundance of bacterial order and families of the phylum Bacteroidota, which differed significantly between the Hp-infected and non-infected mice groups. Unpaired t-test with Welch’s correction was used to test for statistical significance of the difference, *P < 0·05, **P < 0·01, ***P < 0·001.

Given that the altered microbiota in aged mice exhibited reduced responsiveness to Hp infection, we focused on specific bacterial groups within the cecal microbiota that were responsible for the age-related diminishment (Fig. 4E, F). By examining the relative abundance of representative bacterial taxa that exhibited significant changes upon Hp infection, we compared their differential responses in young and aged mice. The bacterial families comprising the cecal microbiota of the mouse groups were relatively consistent; the major taxa included Lachnospiraceae, Muribaculaceae, Ruminococcaceae, Lactobacillaceae, and Prevotellaceae (Fig. 5A). In order to identify bacterial groups that specifically responded to Hp infection, we performed statistical analyses of cecal microbiota between Hp-infected and non-infected groups of young and aged mice. The ratio of the bacterial phyla Bacillota (formally Firmicutes) and Bacteroidota (formally Bacteroidetes), i.e., F/B ratio, has been used an index for gut microbiota composition^34^. In our statistical analysis, Hp-infected young mice showed a significantly lower F/B ratio, whereas this was not observed in the aged mouse groups (Fig. 5B). The specific alteration of cecal microbiota following Hp infection is therefore characterized by an increased relative abundance of the phylum Bacteroidota and a decreased abundance of Bacillota in young mice; however, these changes were less pronounced in the aged mouse groups (Fig. 5B). Statistical analyses of changes in relative abundance of lower-level bacterial taxa in each mouse group have identified bacterial groups responsible for the specific alterations of cecal microbiota in young and aged mice. The relative abundance of the order Clostridiales (Bacillota), which was the most abundant bacterial order in the cecal microbiota of mice (Fig. S3), significantly decreased upon Hp infection in young mice, a change not observed in aged mice (Fig. 5C). Lachnospiraceae and Clostridiaceae likely contributed to the decrease in Clostridiales, as they exhibited similar profiles in their relative abundance (Fig. 5C). The relative abundance of Bacteroidales (Bacteroidota) clearly indicated that the lowered F/B ratio upon Hp infection in young mice is attributed to the increase of this taxon (Fig. 5D). At the family level, Odoribacteriaceae, Prevotellaceae, and Rikenellaceae were likely responsible for the significant increase of Bacteroidales (Fig. 5D). We also identified multiple bacterial genera (*Clostridium*, *Odoribacter*, *Prevotella*, *Paraprevotella*, *Sporofaciens*, and *Turicibacter*) that responded to Hp infection in the young mouse groups but not in the aged mouse groups (Figs. S4, S5). While not statistically significant, the relative abundance of *Bifidobacterium* (*Bifidobacterium pseudolongum*), which represents a major bacterial taxon in the intestines of young mice, decreased in the infected young mice (Fig. S5). Overall, bacterial groups that exhibited significant changes in relative abundance in response to Hp infection in young mice demonstrated reduced responsiveness in aged mice.

## Discussion

Effective activation of type 2 immune responses is essential for the successful expulsion of gastrointestinal nematode parasites. Up-regulation of marker genes for type 2 immune responses, including the genes encoding IL-4, IL-13, IL-25, and Arg1, was observed following Hp infection in young (3-month-old) mice (Fig. 1), which plays a crucial role in the initiation of the host’s defense mechanism against nematodes invading the mucosal sites of the small intestine^35,36^. Our current study corroborates our previous findings that the type 2 immune response is significantly impaired by aging^6^. In 18-month-old aged mice, type 2 cytokines are down-regulated and mucus production is suppressed, which ultimately resulted in the failure of nematode expulsion^6,7^. Since the number of CD4 + T cells—the lymphocytes responsible for type 2 immune protection against nematodes—is consistent between the infected and non-infected mice^6,7^, impairment of lymphocyte activation rather than migration has been attributed to the unsuccessful parasite clearance. Also, IFNγ, whose gene expression was unaffected by Hp infection in young mice but up-regulated in aged mice (Fig. 1), is likely involved in the suppression of type 2 cytokines because this cytokine is known to function antagonistically^37^. Elevated IFNγ levels observed in aged mice may be linked to age-associated inflammation and alterations in the gut microbiota, both of which can impair the proper differentiation and activation of Th2 cells.

As has been documented in a large number of studies, intestinal bacteria and their metabolites play key roles in the activation of immune cells^13,38^. Commensal gut microbiota, which usually maintain stable and resilient microbial communities, can uphold immune homeostasis by constantly “training” immune cells and directly or indirectly controlling host gene expression^39^. Such homeostatic microbiota produce consistent amounts of functional metabolites, such as SCFAs, which have been widely used as indicators of gut microbiota health^40,41^. In the current study, elevated levels of SCFAs (Fig. 2) and gene expression of G protein-coupled receptors (GPR41 and GPR43; Fig. 3) were observed in Hp-infected young mice, which suggests that the protective responses to parasite infection may involve the activities of gut microbiota. Both GPR41 and GPR43 regulate lymphocyte differentiation and cytokine production, and their gene expression and activities are enhanced by SCFAs produced by gut microbiota^42,43^. GPR41 and GPR43 have been reported to modulate type 2 immune responses primarily by suppressing the activity of mast cells, eosinophils, and ILC2s, which are key effector cells involved in type 2 immunity, including helminth defense^42,44^. In response to Hp infection, activation of these receptors stimulates cytokine production and enhances regulatory functions. Since Hp infection itself increases SCFA levels in the intestine^45^synergistic promotion of GPR signaling further enhances type 2 immunity, and such cascades are considered important for effective host defense against helminths.

Previous studies have indeed documented the co-occurrence of microbiota alterations and increased SCFA production following infection with gastrointestinal parasite nematodes^22,23^. In line with this, the impaired immune responses observed in aged mice —characterized by lowered levels of SCFA production and reduced GPR41/GPR43 expression —may be attributed to age-dependent fluctuations in gut microbiota, which appear to respond differently to Hp infection and its metabolites. Although age-related changes in the responsiveness of GPR41/GPR43 remain poorly understood, our data (Fig. 2) show a non-significant trend toward increased SCFA concentrations with age, yet no corresponding change in GPR41/43 gene expression. This suggests a potential attenuation of receptor responsiveness to SCFAs in aged individuals. While a decline in the number of epithelial cells expressing these receptors could contribute to this phenomenon, direct evidence is still lacking.

Our current finding highlight a markedly diminished responsiveness of the gut microbiota to Hp infection in aged mice. The gut microbiota are susceptible to age-related dysbiosis, which may lead to the loss of specific bacterial taxa or microbial functions that are responsive to Hp infection. This loss could impair the proper activation of type 2 responses necessary for effective nematode expulsion. The cecal microbiota of 18-month-old aged mice, both infected and non-infected, showed typical signatures of age-dependent dysbiosis characterized by excessive individual variability (Fig. 4A–D and Supplementary Fig. S2), which is often described as a diminishment of commensal bacterial members and an overrepresentation of minor bacterial groups such as Proteobacteria^46,47^. Commensal gut microbiota in young mice profoundly responded to Hp infection (Fig. 4), with a significant decrease in the ratio of Bacillota (formerly Firmicutes) to Bacteroidota (formerly Bacteroidetes), i.e., the F/B ratio, observed (Fig. 5). Similar results were reported in a previous study^48^and the current findings corroborate the usefulness of the F/B ratio of gut microbiota as an indicator of Hp infection. A decreased F/B ratio reflects a relative increase in Bacteroidota, which are enriched in propionate producers, while Firmicutes are more associated with butyrate production while both contribute to acetate production^49^. This aligns with our observation of a trend toward higher amount of propionate than butyrate levels in Hp-infected young mice (Fig. 2). Hp-induced type 2 immune responses alter mucus and antimicrobial peptide expression, which may favor the proliferation of Bacteroidota. Our findings suggest that this effect is likely suppressed in aged mice, where type 2 immune responses are diminished.

Our study further attributes the alteration in the F/B ratio to shifts in specific bacterial groups, whose responsiveness is diminished in aged mouse groups (Fig. 5). Lachnospiraceae, which represent predominant bacterial taxa in animal gut microbiota, significantly decreased upon Hp infection in young mouse groups, while multiple bacterial families of Bacteroidales, particularly Odoribacteraceae and Prevotellaceae, significantly increased (Fig. 5). Lachnospiraceae have been reported to suppress Th2 responses by inducing dendritic cell TGF-β production, so reduced levels of Lachnospiraceae may increase Th2 activity during Hp infection^50^. Lachnospiraceae are also known as important producers of butyrate, which are involved in maintaining gut barrier integrity and regulating inflammation. Their reduction during helminth infection may have consequences for gut health and immune regulation. On the other hand, there has been no evidence so far regarding how Bacteroidales, including Odoribacteraceae and Prevotellaceae, are involved in Hp infection and type 2 responses. Also, the immunomodulatory effects of their major metabolite, propionate, on type 2 immunity remain entirely uncharacterized. However, both Odoribacteraceae and Prevotellaceae are thought to be associated with health and disease profiles, as well as aging. Prevotellaceae are mainly linked to proinflammatory Th17 responses, while Odoribacteriaceae are believed to be associated with anti-inflammatory and regulatory effects^51,52^. Intriguingly, a strain of Odoribacteraceae has been reported to be an important producer of secondary bile acids, which are known for their antimicrobial effects against Gram-positive bacteria and potentially modulating type 2 immune responses by acting as metabolic signals that influence Th2 cell function^53,54^.

Overall, our findings suggest that age-related declines in both microbiota plasticity and host responsiveness to Hp infection are mediated by the complex interaction within the gut microbiota–helminth–host immune axis. This complexity poses significant challenges for addressing age-related immune impairment, suggesting that simply modulating host gene expression through the gut microbiota manipulation, or vice versa, is unlikely to provide an adequate therapeutic solution. Given the critical, bidirectional regulation between the gut microbiota and immune cells—which together shape both local and systemic immune homeostasis—elucidating the mechanistic contributions of the microbiota is essential for understanding and potentially mitigating age-associated immune decline. Notably, these mechanisms may influence not only resistance to gastrointestinal nematodes but also susceptibility to a wide range of pathogens^25,55^. The heightened vulnerability of elderly individuals to certain infectious diseases, particularly those involving type 2 immune responses, may in part be attributable to similar underlying processes.

In conclusion, our current study highlights the importance of gut microbiota responsiveness during nematode infection for the effective activation of type 2 immune responses. These findings underscores the critical role of the gut microbiome in age-related impairment of parasite clearance. Future research should elucidate the molecular mechanisms underlying reduced microbiota responsiveness in aging—such as the roles of microbial metabolites and interacting host factors—to provide insights for the development of clinical interventions to address age-related immune decline by restoring the complex gut ecology.

## Methods

### Ethical statement

The authors confirm that all animal experiments were conducted in accordance with the ARRIVE guidelines. The animal experimental protocols were evaluated and approved by the Institutional Ethics Commission for Animal Research of Miyagi University (Miyagi University Environmental and Safety Committee on Experimental Animal Care and Use)　 and we confirm that all methods were performed in accordance with the relevant guidelines and regulations.

### Nematode infection of mice and sampling

Female BALB/c mice were purchased from Japan SLC, Inc. (Shizuoka, Japan) and used for all experiments. The animals were divided into two groups: young (3 months old) and old (18 months old).These age groups were selected based on previous studies that are widely accepted in the field as representative models for studying aging. They are commonly employed to ensure relevance to human aging: 3 months, corresponding to young adults (20–30 years) in humans, and 18 months, corresponding to aged adults (60–70 years) in humans. The mice were housed under conventional conditions with food and water provided ad libitum, and the cages were maintained on a 12/12-hour light/dark cycle until they reached the designated ages. The animals’ environment was maintained at 22 °C ± 1.5 °C with a relative humidity of 55% ± 5%. Mice were inoculated orally with 200 L3 larvae of *Heligmosomoides polygyrus* (Hp), while control mice received saline orally during the same period. On Day 8, the mice were anesthetized by intraperitoneal injection of a cocktail of three anesthetic agents (medetomidine at 0.3 mg/kg, midazolam at 4 mg/kg, and butorphanol at 5 mg/kg), sacrificed by cervical dislocation, and tissue samples from the small intestine and cecum were collected.

### Gene expression analysis

Total RNA extraction from the small intestinal tissue were performed using TRI Reagent^®^ (Molecular Research Center, Inc., Frederic, OH, USA) according to the manufacturer’s instructions. RNA levels were measured using a NanoDrop spectrophotometer (Thermo Scientific, Wilmington, DE, USA), and cDNA was synthesized with random primers and SuperScript II (Life Technologies, Inc., Frederic, MD, USA). Primer sequences for the genes encoding IL-4, IL-13^56^, IL-25^26^ and Arginase 1 (ARG1)^57^ have been described previously. Primer sequences for GPR41 and GPR43 were designed using primers from the national Center for Biotechnology Information (NCBI) database, with the following sequences: GPR41. forward: 5’-AGGCTGGTCTGGTCAGTGTA-3’, reverse: 5’-CATTGGTCCCCTGGCTGTAG-3’. GPR43. forward: 5’-GGTGTGCTTTGGACCCTACA-3’, reverse: 5’-AACTGAACACCACAGCCTCC-3’.

Real-time PCR was performed using the Brilliant SYBR Green QPCR Master Mix III (Stratagene, La Jolla, CA, USA) with an AriaMx system (Agilent Technologies, Santa Clara, CA, USA). The amplification conditions were as follows: 95 ℃ for 3 min, followed by 40–50 cycles of 95 ℃ for 5 s and 60 ℃ for 20 s. Fluorescence signals measured during the amplification were processed afterward. Ribosomal RNA primers were used as an internal control, and all data were normalized to constitutive rRNA values. Quantitative differences between the groups were analyzed using the AriaMx software (Agilent Technologies).

### Short chain fatty acid (SCFA) measurement by gas chromatography

Mice cecal contents were homogenized in a threefold volume of distilled water using a bead crusher at 3,000 rpm for 60 s (µT-12; TAITEC, Japan). The homogenates were centrifuged at 4 °C, 1,000 rpm for 12 min and the supernatants were transferred to sealed vials and analyzed for SCFAs by gas chromatography equipped with a flame-ionization detector (model 6890 N, Agilent, Palo Alto, CA). Separations were performed on a capillary column (0.25 mm x 30 m x 0.25 μm, DB-FAT WAX UI, Agilent, Palo Alto, CA). The initial column temperature was 100 °C programmed to increase at 7 °C/min to 260 °C, where it was maintained at 260 °C for 5 min. Injector and detector temperatures were 280 °C. Helium was used as the carrier gas at a flow rate of 1 ml/min. Standards included acetic acids, butyric acids, propionic acid (Wako Pure Chemical Industries, Japan).

### 16S rRNA gene analyses of cecal microbiota

DNA was extracted from the cecal samples using QIAamp PowerFecal Pro DNA Kit (QIAGEN, Tokyo, Japan) following manufacturer’s instructions. The quality and quantity of the extracted DNA samples were evaluated using a NanoDrop spectrophotometer (Thermo Scientific, Wilmington, DE). 16S rRNA gene amplicon sequencing (V4 region, 2 × 150 paired-end) was performed using MiSeq platform (Illumina) at Seibutsu Giken Inc. (Japan). The raw FASTQ sequencing reads were paired-ended, trimmed, filtered and clustered into OTUs using EzBioCoud 16S database version PKSSU4.0 implemented in the 16S Microbiome Pipeline in the EzBioCloud^58^. Statistical diversity analyses were performed using Microbiomeanalyst©^59^.

### Statistical analysis﻿

Statistical analyses of gene expression levels and amounts of short-chain fatty acids (SCFAs) were performed using GraphPad Prism (version 10.4.1, GraphPad Software, San Diego, CA). Statistical comparisons between the age (young vs. aged) and infection status (infected vs. noninfected) were conducted using two-way analysis of variance (ANOVA), followed by Tukey’s post hoc test. Differences were considered statistically significant at *p* < 0.05. Gene expression data are presented as mean ± standard error (SE) for each treatment group. Each experimental group included appropriate time- and age-matched controls (*n* = 5–6 per group).

For cecal microbiota analyses, alpha and beta diversity analyses were performed using MicrobiomeAnalyst©^59^. For alpha diversity, metrics including observed species richness, Chao1, ACE, Shannon, and Simpson indices were calculated to assess within-sample diversity. Beta diversity was evaluated using distance matrices based on Bray-Curtis dissimilarity and visualized with principal coordinates analysis (PCoA). Statistical significance of alpha diversity differences between groups was assessed using the non-parametric Kruskal-Wallis test. Differences in beta diversity were evaluated using permutational multivariate analysis of variance (PERMANOVA) based on the distance matrices using the default setting of MicrobiomeAnalyst©^59^ platform where p-values < 0.05 were considered statistically significant. Rarefaction was applied to normalize sequencing depth across samples before diversity calculations. To compare the relative abundance of different bacterial taxa between age groups (3 months vs. 18 months) and infection status (infected vs. non-infected), statistical analysis was performed using an unpaired t-test with Welch’s correction implemented in Prism GraphPad Prism, version 5.02 (GraphPad Software Inc., San Diego, CA) (*P < 0·05, ***P < 0·001, ****P < 0·0001).

## Supplementary Information

Below is the link to the electronic supplementary material.

Supplementary Material 1

## Data Availability

The raw amplicon sequence data files were deposited in the NCBI sequence read archive (SRA) database under BioProject accession number PRJNA1163063 (https://www.ncbi.nlm.nih.gov/bioproject/PRJNA1163063).
