# Supplementary material for: Aging impairs type 2 immune responses to nematodes associated with reduced gut microbiota responsiveness
Source: Sci Rep. 2025 Aug 26;15:31385. doi: 10.1038/s41598-025-16730-x (PMC12381008; doi:10.1038/s41598-025-16730-x)
Supplement: Supplementary file 1 — Supplementary Material 1 [file 41598_2025_16730_MOESM1_ESM.pdf]

Fig. S1.

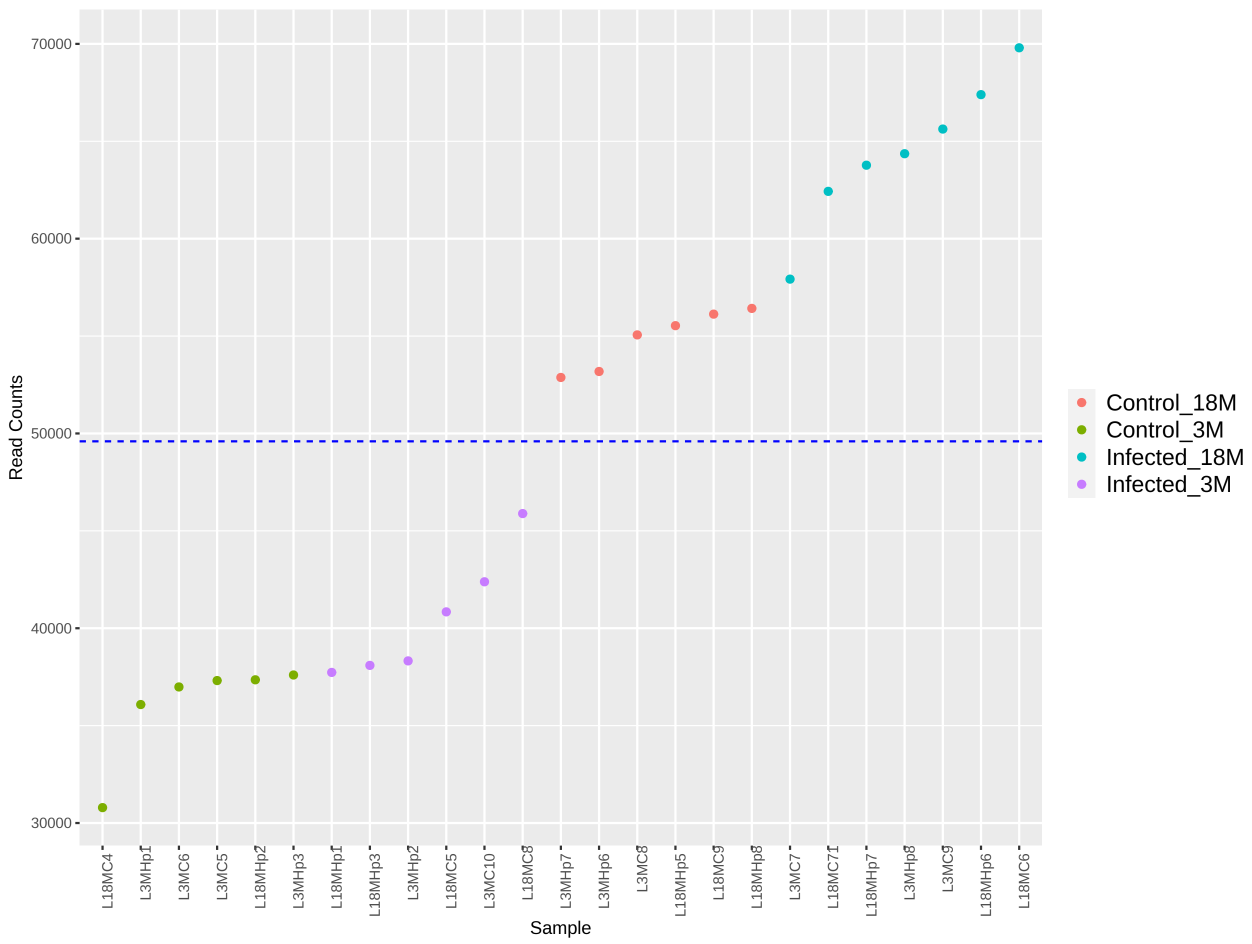

Supplementary Fig. S1. Number of sequencing reads obtained from each mouse cecal samples

Fig. S2

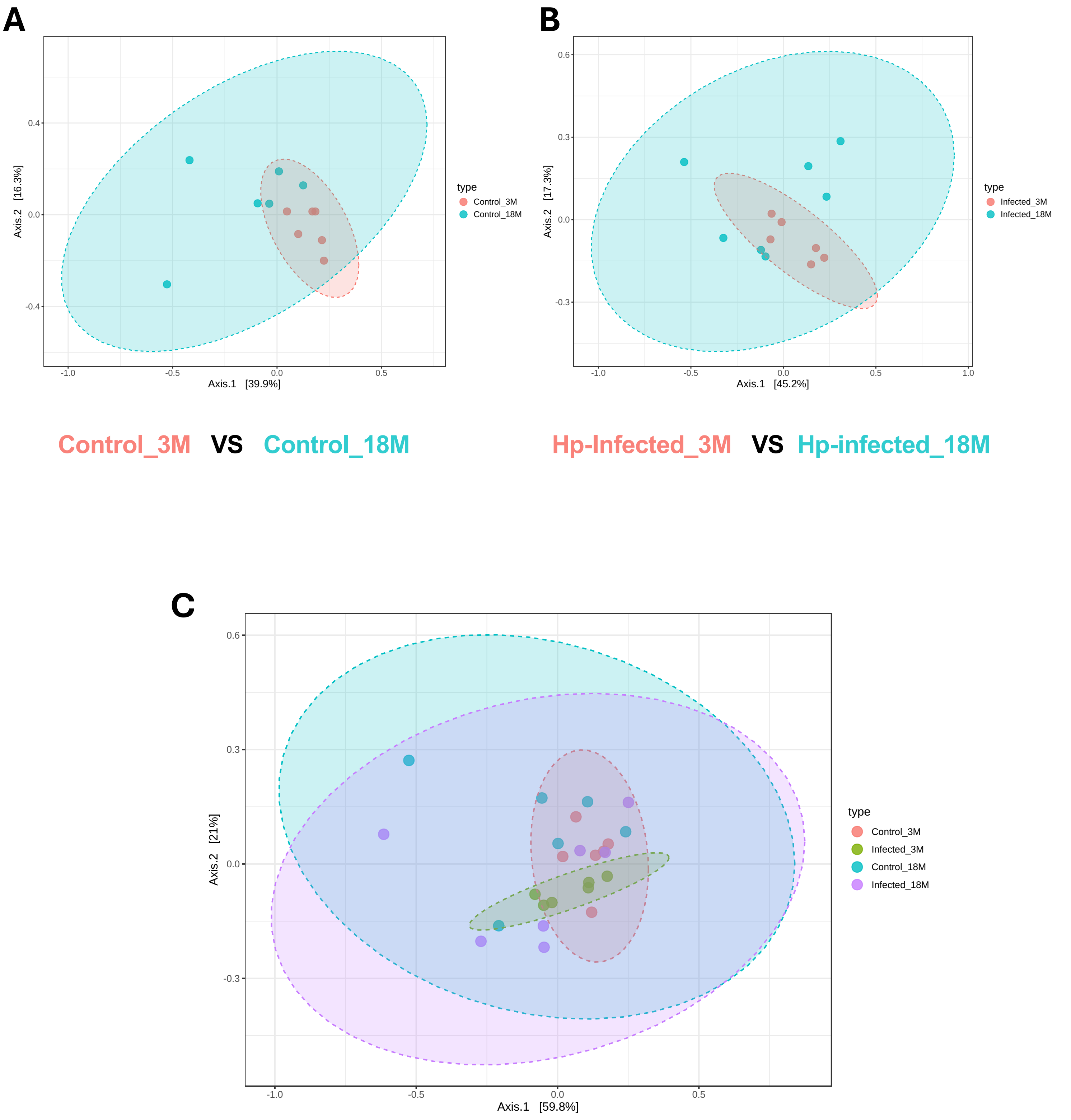

Supplementary Fig. S2. Beta diversity of species-level bacterial community in cecal microbiota of young (3 M) and aged (18 M), Hp-infected and non-infected mice . Comparison between the samples were performed by principal coordinate (PCoA) analysis based on Bray-Curtis dissimilatory index.

Fig. S3

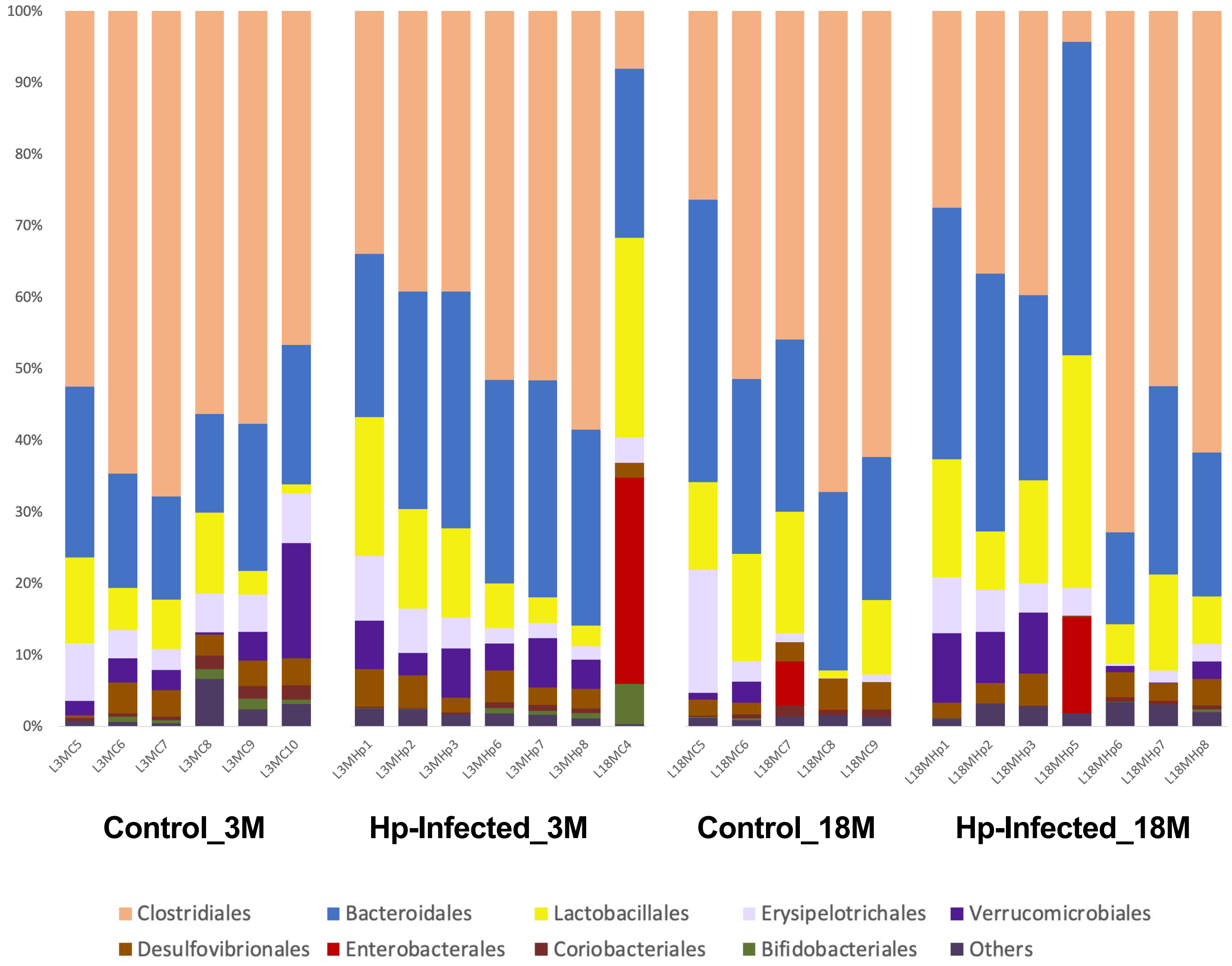

Supplementary Fig. S3. Order-level bacterial composition of the mice cecal microbiota.

Fig. S4

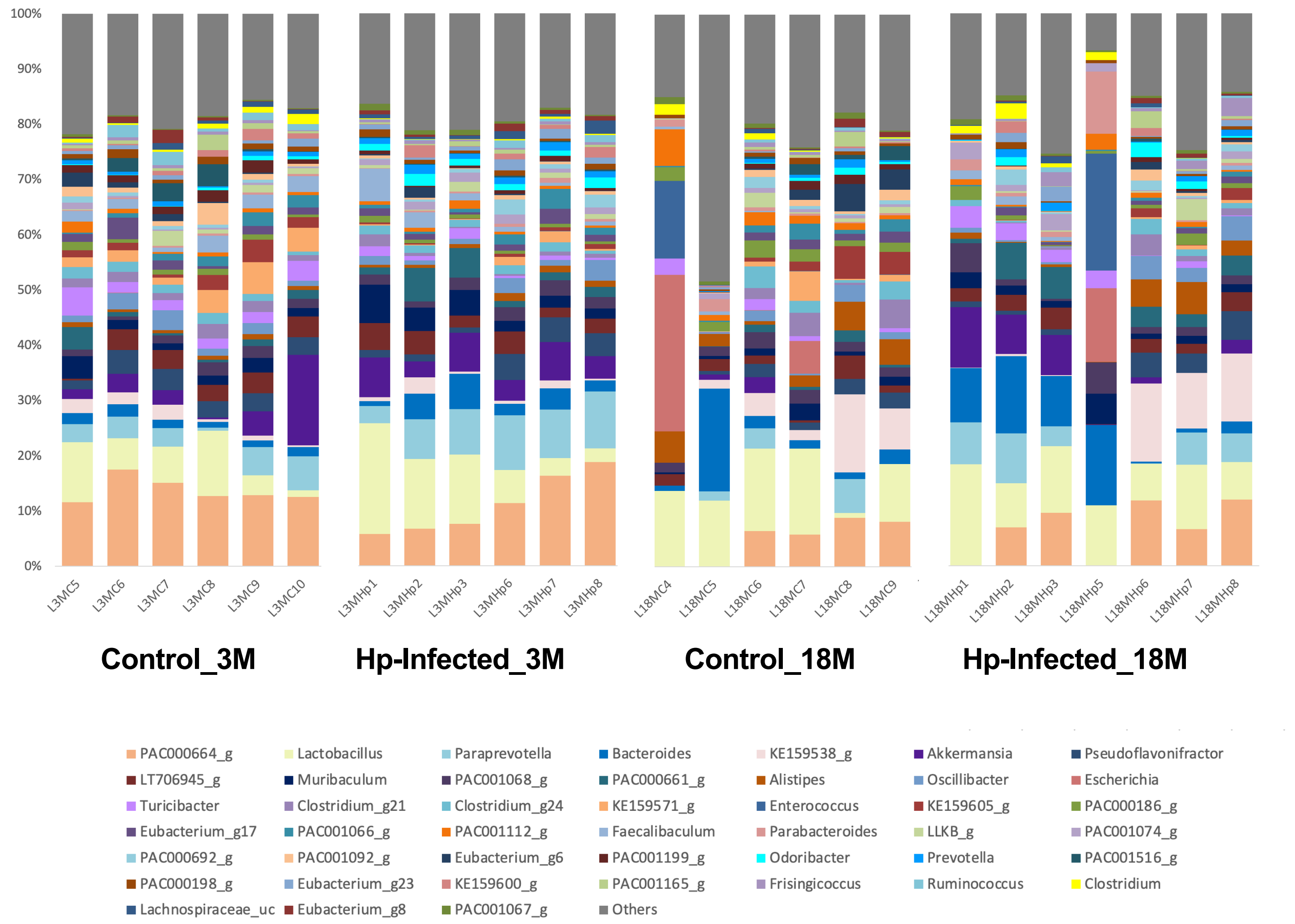

Supplementary Fig. S4. Genus-level bacterial composition of the mice cecal microbiota. The taxonomic affiliation is based on the EzBioCloud database.

Fig. S5

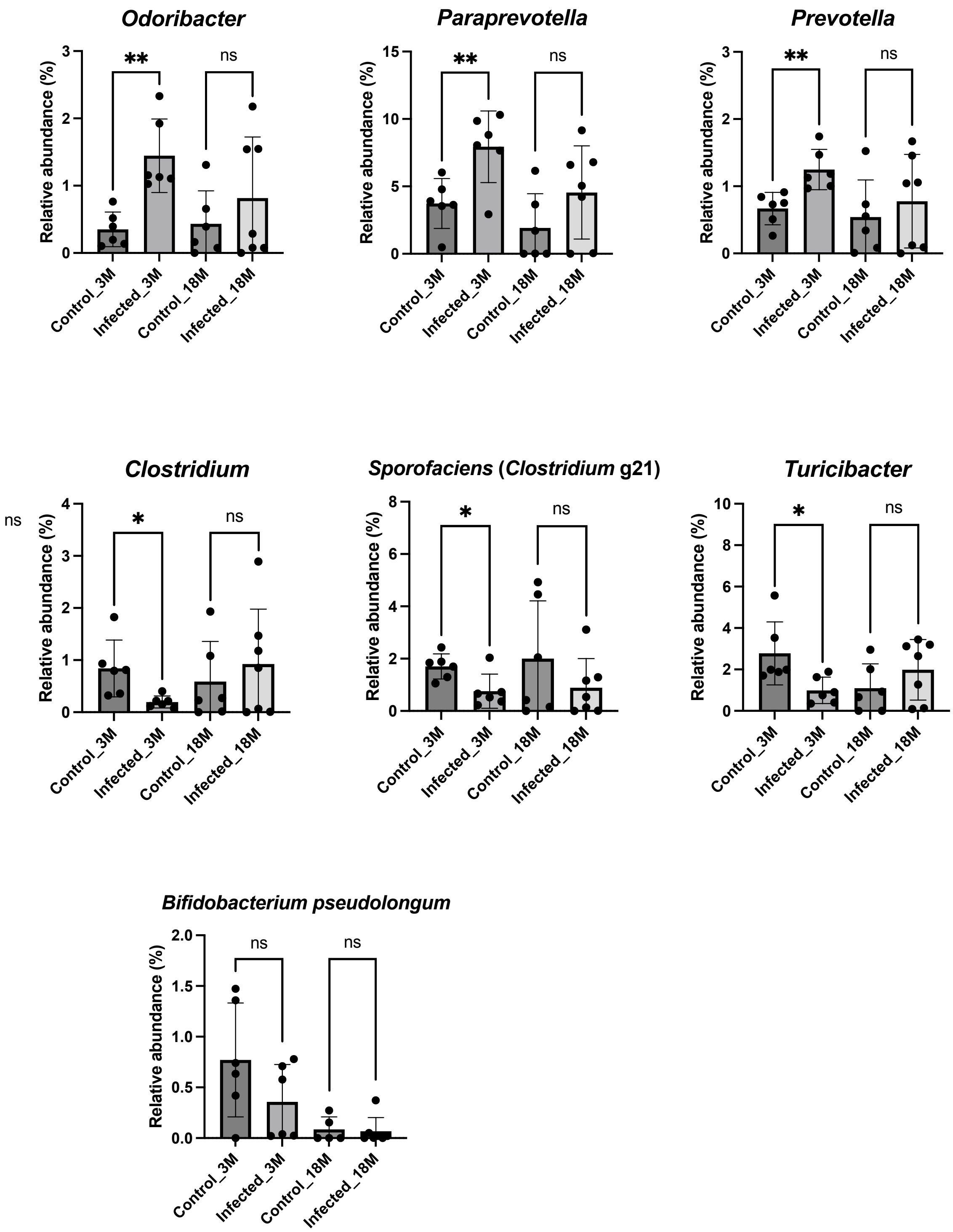

Supplementary Fig. S5. Relative abundance of bacterial genera, which differed significantly between the Hp-infected and non-infected mice groups. Unpaired t-test was used to test for statistical significance, *P < 0·05, **P < 0·01
